# Genome-wide identification and characterization of members of the LEA gene family in *Panax notoginseng* and their transcriptional responses to dehydration of recalcitrant seeds

**DOI:** 10.1186/s12864-023-09229-0

**Published:** 2023-03-17

**Authors:** Jin-Shan Jia, Na Ge, Qing-Yan Wang, Li-Ting Zhao, Cui Chen, Jun-Wen Chen

**Affiliations:** 1 College of Agronomy & Biotechnology, Yunnan Agricultural University, Kunming, 650201 Yunnan China; 2 The Key Laboratory of Medicinal Plant Biology of Yunnan Province, Yunnan Agricultural University, Kunming, 650201 Yunnan China; 3 National & Local Joint Engineering Research Center On Germplasm Innovation & Utilization of Chinese Medicinal Materials in Southwestern China, Yunnan Agricultural University, Fengyuan Road, Panlong District, Kunming, 650201 Yunnan China

**Keywords:** LEA, Expression patterns, Dehydration stress, Recalcitrant seeds, *Panax notoginseng*

## Abstract

**Background:**

Late embryogenesis abundant (LEA) proteins play an important role in dehydration process of seed maturation. The seeds of *Panax notoginseng* (Burkill) F. H. Chen are typically characterized with the recalcitrance and are highly sensitive to dehydration. However, it is not very well known about the role of LEA proteins in response to dehydration stress in *P. notoginseng* seeds. We will perform a genome-wide analysis of the LEA gene family and their transcriptional responses to dehydration stress in recalcitrant *P. notoginseng* seeds.

**Results:**

In this study, 61 LEA genes were identified from the *P. notoginseng* genome, and they were renamed as *PnoLEA*. The *PnoLEA* genes were classified into seven subfamilies based on the phylogenetic relationships, gene structure and conserved domains. The *PnoLEA* genes family showed relatively few introns and was highly conserved. Unexpectedly, the LEA_6 subfamily was not found, and the LEA_2 subfamily contained 46 (75.4%) members. Within 19 pairs of fragment duplication events, among them 17 pairs were LEA_2 subfamily. In addition, the expression of the *PnoLEA* genes was obviously induced under dehydration stress, but the germination rate of *P. notoginseng* seeds decreased as the dehydration time prolonged.

**Conclusions:**

We found that the lack of the LEA_6 subfamily, the expansion of the LEA_2 subfamily and low transcriptional levels of most *PnoLEA* genes might be implicated in the recalcitrant formation of *P. notoginseng* seeds. LEA proteins are essential in the response to dehydration stress in recalcitrant seeds, but the protective effect of LEA protein is not efficient. These results could improve our understanding of the function of LEA proteins in the response of dehydration stress and their contributions to the formation of seed recalcitrance.

**Supplementary Information:**

The online version contains supplementary material available at 10.1186/s12864-023-09229-0.

## Background

Nowadays, LEA gene family has been identified in rice (*Oryza sativa*) [1], maize (*Zea mays*) [2], *Brassica napus* [3], wheat (*Triticum aestivum*) [4] and *Arabidopsis thaliana* [5]. LEA proteins are found in large numbers in plant species, for example, *A. thaliana* has 51 members [5], *B. napus* has 108 [3] and wheat has 281 [4]. The number of LEA genes is different across species and the diversity might be related to the response of plants to abiotic stresses. The 26 *MeLEA* genes were identified in cassava (*Manihot esculenta* Crantz) and were observed to respond to multiple abiotic stresses, and H_2_O_2_ and ABA signaling [6]. The transgenic *A. thaliana* and foxtail millet (*Setaria italica*) plants overexpressing *SiLEA14* showed higher tolerance to salt and osmotic stress than the wild type (WT) [7]. The 33 *CsLEA* genes have been identified in the genome of the recalcitrant seed of tea tree, and they are closely related to the response to low temperature and dehydration stresses in tea tree (*Camellia sinensis*) [8]. Currently, the LEA gene family have become a popular research topic in plant response to stress.

Late embryogenesis abundant (LEA) proteins is firstly found in cotton (*Gossypium hirsutum*) seeds [9]. Cotton seeds significantly accumulate LEA proteins when they mature and dehydration in order to protect them from damage [9]. The expressions of LEA genes have been recorded in different tissues, including seeds, roots, stems and buds [10]. For example, the expression of most LEA genes showed different tissue-specificity in maize [2]. The *SmLEA* genes of *Salvia miltiorrhiza* are specifically expressed in distinct tissues, and most of them are up regulated when *Salvia miltiorrhiza* is under drought conditions [11]. LEA proteins are generally classified into eight subfamilies based on the similarity of sequences and specific conserved domains, including LEA_1, LEA_2, LEA_3, LEA_4, LEA_5, LEA_6, dehydrin (DHN) and seed maturation protein (SMP) [12]. The molecular weight of most LEA proteins ranges from 10 to 30 kDa. LEA proteins are composed with glycine and other hydrophilic amino acids, which are highly hydrophilic and heat stable and play a role in stabilizing cell membranes, molecular barriers, ion binding and antioxidant in plants under stress [13]. LEA proteins are protectors of cell membranes and biomolecules, and they stabilize the structure of other proteins and cell membranes by forming dense hydrogen bonds [14, 15]. LEA proteins retargeting intracellular water molecules, binding salt ions, and eliminating active oxygen radicals accumulated in cells due to dehydration [16]. In addition, LEA proteins can combine with misfolded proteins through molecular chaperones that stabilize denatured proteins and promote their refolding [17]. The overexpression of the Group LEA_4 protein from *B. napus* considerably improve abiotic stress tolerance including salt stress and drought stress in transgenic *A. thaliana* plants [18]. The overexpression of the *ShDHN* gene enhances the tolerance of tomato (*Solanum lycopersicum*) to abiotic stresses [19]. Drought tolerance is enhanced through protecting embryos and endosperm from water deficiency in transgenic *A. thaliana* plants with *MdoDHN11* overexpression [20]. Therefore, LEA proteins are essential to the process of obtaining dehydration tolerance.

Seeds could be divided into orthodox and recalcitrant according to their storage characteristics and desiccation tolerance [21, 22]. Recalcitrant seeds maintain a high water content when they mature and fall off, and it is sensitive to dehydration and low temperature during the growth and development process [23]. The germination rate decrease from 92 to 50% when the water content of recalcitrant *Ginkgo biloba* seeds is reduced from 48% to 40.1% [24]. Similarly, recalcitrant *Saraca asoca* seeds show an initial water content of 56.8% and are completely inactivated when water content is reduced to between 11 and 17% [25]. The accumulation of LEA proteins play an important role in the acquirement of dehydration tolerance [26–28]. LEA proteins are hydrophilic and they create a protective membrane around the cellular internal structure and macromolecules, consequently making the seeds confer dehydration tolerance [29]. High accumulation of LEA proteins has been observed in the orthodox maize seeds during maturation dehydration [30]. The lack of LEA protein is found in dehydration-sensitive recalcitrant *Avicennia marina* seeds [31]. The deficiency of LEA proteins may be an essential reason for its susceptibility to dehydration in the recalcitrant seeds of chestnut bean tree (*Castanospermum australe*) [32]. Gene expression of antioxidant enzymes and LEA proteins are down-regulated in recalcitrant tea tree during dehydration process [33]. However, it is still unclear whether the lack of LEA proteins causes the dehydration sensitivity of the recalcitrant seeds.

*Panax notoginseng* (Burk.) F. H. Chen (Sanqi in Chinese), is a perennial herb of the family of Araliaceae [34]. The *Panax notoginseng* seeds belong to the group of morphophysiological dormancy (MPD) type, and moreover it has been typically characterized by the recalcitrant trait that show a high water content at postharvest after-ripening process [35]. The seeds need to undergo about 45 ~ 60 days of after-ripening process before the germination [36]. It is extremely unfavorable for the storage of *P. notoginseng* seeds with dehydration sensitivity and dormancy. Slow dehydration is more harmful to *P. notoginseng* seeds than rapid dehydration [37]. Membrane peroxidation and the reduced activity of antioxidant enzyme are one of the important reasons for the dehydration sensitivity of *P. notoginseng* seeds [35]. Recently, RNA-Seq analysis showed that the *LATE EMBRYOGENESIS ABUNDANT PROTEIN DC3* and *DEHYDRIN9* may be involved in the dehydration sensitivity of *P. notoginseng* seeds at different after-ripening stages [38]. Our previous study has found that the lack of LEA proteins in embryos may be a key factor in the dehydration sensitivity of recalcitrant *P. notoginseng* seeds [39]. However, the identification of the LEA gene family in recalcitrant seeds of *P. notoginseng* has not been performed in the context of whole genome and thus it is not very well known about the functions of the PnoLEA proteins, especially in the response to dehydration stress.

In our study, the LEA genes would be identified from the genome of *P. notoginseng*, and we analyzed the gene structure, conserved domains, phylogenetic relationship, chromosomal location and duplication event. In addition, we analyzed the expression of the *PnoLEA* genes in distinct tissues and the response to dehydration stress. These results would improve our understanding of the *PnoLEA* genes family, and this study would provide a new insight for the functions of LEA proteins of recalcitrant seeds under dehydration stresses.

## Results

### Identification of *PnoLEA* genes in the *P. notoginseng*

We identified 61 LEA genes in the *P. notoginseng* genome by combining HMMER and local BLAST methods (Table 1). We renamed each *PnoLEA* genes according to its localization on the *P. notoginseng* chromosome. Based on conserved domains, *PnoLEA* genes were divided into seven subfamilies and the LEA_6 subfamily was not identified in *P. notoginseng* genome. The LEA_2 subfamily had 46 (75.4%) members and was the largest number of subfamily members. The LEA_1 subfamily, DHN subfamily and SMP subfamily contained 2, 6 and 4 members, respectively. The LEA_3 subfamily, LEA_4 subfamily and LEA_5 subfamily were only one gene member. The 61 PnoLEA proteins showed different physicochemical properties (Table 1). The 61 *PnoLEA* genes encoded polypeptides ranging from 106 to 865 amino acids, and predicted molecular weights of the 61 PnoLEA proteins range from 10.96706 (*PnoLEA10*) to 100.10171 (*PnoLEA32*) kDa. The 61 PnoLEA proteins predicted the isoelectric points (pI) ranging from 4.65 (*PnoLEA60*) to 10.57 (*PnoLEA18*). The hydropathicity (GRAVY) values of the 61 PnoLEA proteins between -1.35 and 0.389, and the hydropathicity (GRAVY) values of 42 PnoLEA proteins (68.8%) were less than 0. It suggests that most of the PnoLEA proteins were highly hydrophilic. The prediction of subcellular localization indicated that the most PnoLEA proteins were located in the endomembrane system and nucleus, but a few PnoLEA proteins were distributed in the organelle membrane, plasma membrane or chloroplast. All PnoLEA proteins of the DHN subfamily and SMP subfamily were distributed in the nucleus.

**Table 1:** Sequence characteristics and physicochemical parameter of *LEA* genes in the *P. notoginseng* genome

**Name**	**Gene ID**	**Subfamily**	**Amino acid number**	**KDa**	**pI**	** GRAVY**	**Subcellular Localization**
PnoLEA1	Pno01G004215.t1	LEA_2	317	34.38	9.94	-0.319	Chloroplast
PnoLEA2	Pno01G005297.t1	LEA_2	218	24.35	9.77	-0.229	Nuclear
PnoLEA3	Pno01G005299.t1	LEA_2	222	24.92	10.04	-0.228	Chloroplast
PnoLEA4	Pno01G006107.t1	LEA_2	269	30.65	10.23	-0.087	Chloroplast
PnoLEA5	Pno01G006785.t1	LEA_2	236	26.86	9.53	-0.222	Plasma membrane
PnoLEA6	Pno01G006787.t1	LEA_2	202	22.61	10.04	0.151	Chloroplast
PnoLEA7	Pno01G006789.t1	LEA_2	202	22.83	9.94	0.145	Vacuole
PnoLEA8	Pno01G006795.t1	LEA_2	203	23.09	9.64	0.021	Cytoplasmic
PnoLEA9	Pno01G006807.t1	LEA_2	295	33.09	5.59	-0.399	Golgi
PnoLEA10	Pno01G008106.t1	LEA_1	106	10.97	9.26	-0.923	Nuclear
PnoLEA11	Pno01G008452.t1	LEA_2	199	21.78	9.66	-0.025	Chloroplast
PnoLEA12	Pno01G014208.t1	Dehydrin	225	25.17	4.93	-1.25	Nuclear
PnoLEA13	Pno02G003087.t1	Dehydrin	842	94.29	5.25	-0.264	Cytoplasmic
PnoLEA14	Pno02G004186.t1	LEA_2	200	21.59	9.84	0.335	Chloroplast
PnoLEA15	Pno02G005420.t1	LEA_2	310	34.50	9.69	-0.461	Plasma membrane
PnoLEA16	Pno02G006729.t1	SMP	347	37.84	5.98	-0.379	Cytoplasmic
PnoLEA17	Pno02G007168.t1	LEA_2	252	28.40	10.21	-0.113	Cytoplasmic
PnoLEA18	Pno02G009256.t1	LEA_2	251	27.51	10.57	-0.191	Chloroplast
PnoLEA19	Pno02G011491.t1	LEA_2	306	33.94	10.04	-0.288	Chloroplast
PnoLEA20	Pno02G011751.t1	LEA_2	213	23.96	5.94	0.017	Cytoplasmic
PnoLEA21	Pno03G004189.t1	LEA_2	188	20.64	9.37	0.383	Chloroplast
PnoLEA22	Pno03G004228.t1	LEA_2	193	22.22	9.64	-0.291	Extracellular
PnoLEA23	Pno04G002202.t1	LEA_2	257	28.93	10.18	-0.165	Cytoplasmic
PnoLEA24	Pno04G003855.t1	LEA_2	252	28.48	9.9	-0.202	Cytoplasmic
PnoLEA25	Pno04G005051.t1	LEA_2	316	35.77	7.65	0.251	Plasma membrane
PnoLEA26	Pno04G013501.t1	LEA_2	200	21.72	9.33	0.276	Mitochondrial matrix
PnoLEA27	Pno05G001957.t1	LEA_2	224	24.98	9.41	0.05	Cytoplasmic
PnoLEA28	Pno05G003675.t2	LEA_2	258	28.52	10.17	-0.152	Cytoplasmic
PnoLEA29	Pno05G003676.t1	LEA_2	208	23.63	8.91	0.097	Cytoplasmic
PnoLEA30	Pno05G003722.t1	LEA_2	181	19.63	6.1	0.356	Chloroplast
PnoLEA31	Pno05G003884.t1	LEA_2	201	22.96	9.97	-0.066	Chloroplast
PnoLEA32	Pno05G016210.t1	Dehydrin	865	100.10	5.26	-1.323	Nuclear
PnoLEA33	Pno05G017027.t1	LEA_5	113	12.33	9.6	-1.35	Nuclear
PnoLEA34	Pno06G000531.t1	LEA_2	185	20.19	4.72	0.145	Chloroplast
PnoLEA35	Pno06G001741.t1	LEA_2	201	22.50	9.37	-0.012	Chloroplast
PnoLEA36	Pno06G003570.t1	Dehydrin	164	17.59	9.32	-0.818	Nuclear
PnoLEA37	Pno06G003782.t1	LEA_2	208	23.64	8.34	0.015	Cytoplasmic
PnoLEA38	Pno06G004111.t1	LEA_2	227	25.48	9.77	0.025	Cytoplasmic
PnoLEA39	Pno06G012644.t1	SMP	185	18.63	4.8	-0.189	Cytoplasmic
PnoLEA40	Pno06G013284.t1	LEA_2	205	23.25	9.32	0.088	Cytoplasmic
PnoLEA41	Pno06G013403.t1	LEA_2	192	21.38	9.55	-0.173	Cytoplasmic
PnoLEA42	Pno07G003203.t1	LEA_2	522	57.82	8.83	-0.039	Chloroplast
PnoLEA43	Pno07G003687.t1	Dehydrin	135	14.23	9.49	-0.851	Chloroplast
PnoLEA44	Pno07G005621.t1	LEA_2	195	21.18	9.71	0.359	Cytoplasmic
PnoLEA45	Pno08G002349.t1	LEA_2	225	25.21	9.76	0.389	Chloroplast
PnoLEA46	Pno08G002530.t1	LEA_2	257	27.86	10.29	-0.144	Chloroplast
PnoLEA47	Pno08G004154.t1	LEA_2	320	35.55	4.66	-0.355	Cytoplasmic
PnoLEA48	Pno08G004310.t1	LEA_3	179	18.57	5.04	0.101	Endoplasmic reticulum
PnoLEA49	Pno09G001128.t1	LEA_2	210	23.47	9.62	0.12	Cytoplasmic
PnoLEA50	Pno09G001130.t1	LEA_2	240	27.29	9.65	-0.159	Plasma membrane
PnoLEA51	Pno09G001376.t1	LEA_4	180	18.86	5.32	-0.933	Mitochondrial matrix
PnoLEA52	Pno09G002674.t1	LEA_2	211	23.65	9.79	-0.073	Golgi
PnoLEA53	Pno10G009339.t1	LEA_2	211	23.39	9.3	-0.245	Cytoskeleton
PnoLEA54	Pno12G000411.t1	Dehydrin	192	19.89	8.51	-1.2	Nuclear
PnoLEA55	Pno12G009463.t1	SMP	203	21.41	5.66	-0.799	Nuclear
PnoLEA56	Pno13G000968.t1	LEA_1	142	15.71	5.38	-1.151	Nuclear
PnoLEA57	Pno13G001626.t1	LEA_2	153	16.64	5.15	-0.029	Endoplasmic reticulum
PnoLEA58	Pno13G001773.t1	LEA_2	230	25.06	9.49	-0.081	Cytoplasmic
PnoLEA59	Pno14G001256.t1	LEA_2	318	34.56	9.94	-0.376	Chloroplast
PnoLEA60	Pno16G001656.t1	LEA_2	318	35.39	4.65	-0.323	Cytoskeleton
PnoLEA61	Pno16G002334.t1	SMP	116	12.27	6.05	-0.37	Cytoplasmic

### Gene structure and conserved structural domains of PnoLEA proteins

**Fig. 1:**
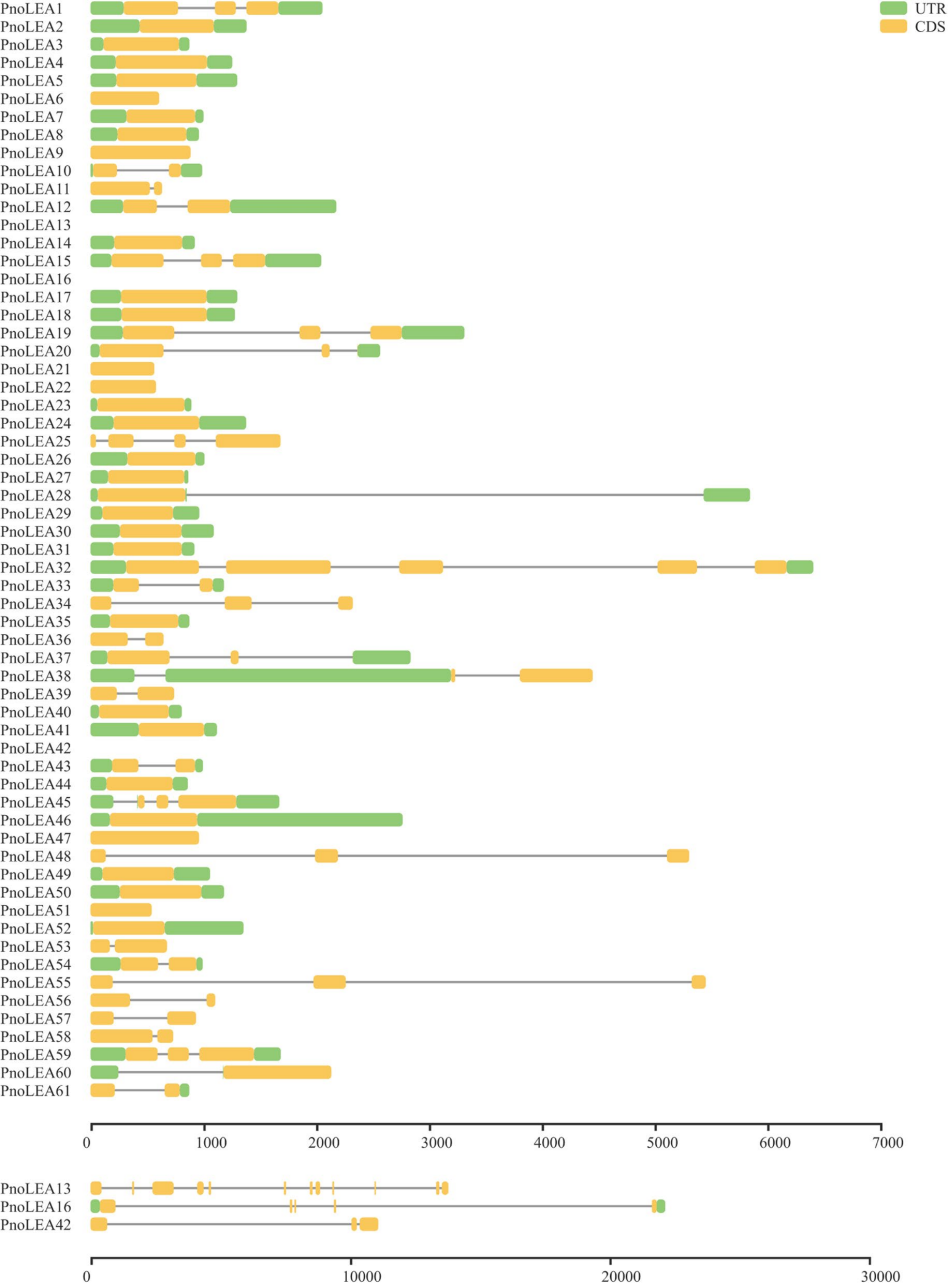
The structure of the *PnoLEA* genes in *P. notoginseng*. The green boxes represent untranslated regions, the yellow boxes represent coding regions, and the lines represent introns

The structure of 61 *PnoLEA* genes was analyzed to reveal their intron and exon characteristics (Fig. 1). The analysis of the structures of *PnoLEA* genes showed that most genes had between 0 and 2 introns. The 51% of *PnoLEA* genes contain no intron, 75% of *PnoLEA* genes contain 0 ~ 1 intron, and only 6% of *PnoLEA* genes have more than 2 introns. The *PnoLEA25* had 3 introns, *PnoLEA16* and *PnoLEA32* had 4 introns, respectively.

**Fig. 2:**
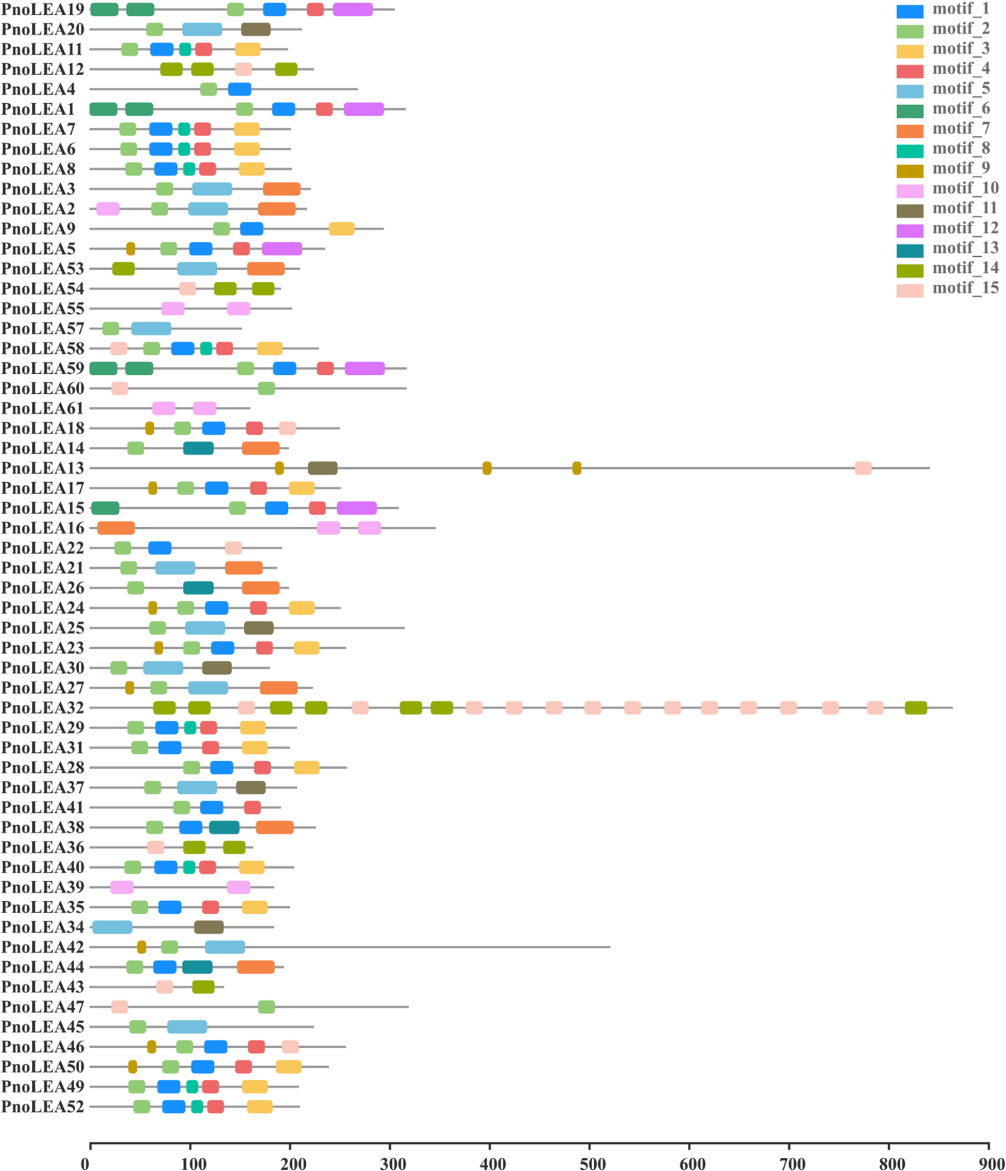
Conserved motifs in PnoLEA proteins. These motifs were identified using Multiple EM for Motif Elicitation (MEME) and different colors boxes represent different motifs. The maximum number of motifs in each sequence is set to 15

The motif characteristics of 56 PnoLEA proteins were analyzed by MEME tool (Fig. 2). The LEA_1 subfamily (*PnoLEA10* and *PnoLEA56*), LEA_3 subfamily (*PnoLEA48*), LEA_4 subfamily (*PnoLEA51*) and LEA_5 subfamily (*PnoLEA33*) had few members, and their motifs were hardly found in other subfamilies, so they were not analyzed together. The members of the same subfamily are similar in the type and number of motifs. Both motif_14 and motif_15 were found in members of the DHN subfamily. All members of the SMP subfamily have motif_10.

### Phylogenetic analyses of the *PnoLEA* genes

**Fig. 3:**
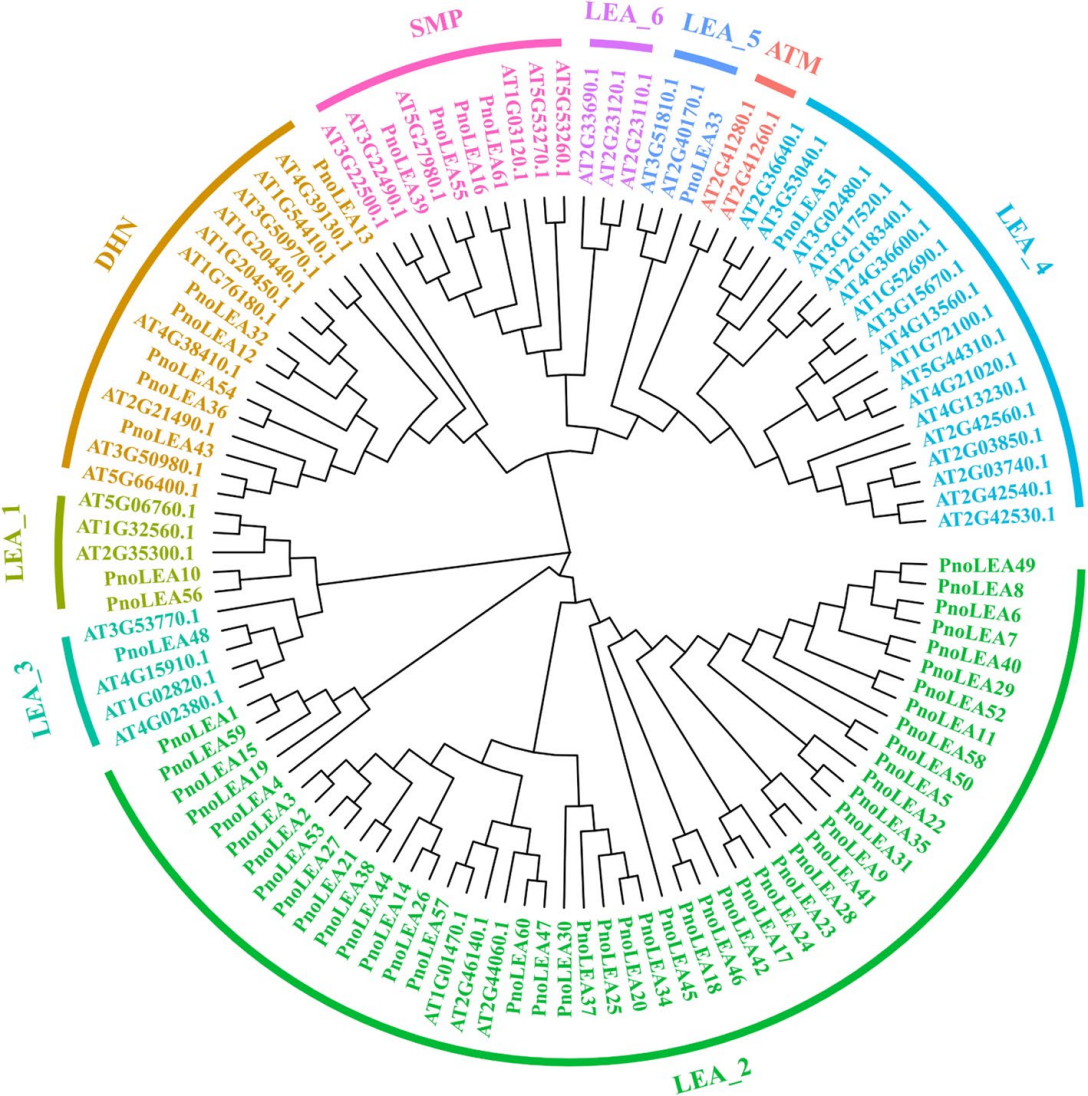
Phylogenetic analysis of LEA proteins in *P. notoginseng* and *Arabidopsis.* Different colors represent different *PnoLEA* genes families. The tree was constructed using MEGA11 by the neighbor-joining (NJ) method based on alignment by MAFFT

To classify the *PnoLEA* genes, a neighbor-joining (NJ) tree was constructed using the protein sequences of the identified 61 PnoLEA and 51 AtLEA (Fig. 3). The LEA families were clustered into nine subfamilies, including LEA_1, LEA_2, LEA_3, LEA_4, LEA_5, LEA_6, DHN, SMP and ATM. The 61 *PnoLEA* genes were divided into seven subfamilies, and the LEA_6 subfamily was not present in the *P. notoginseng* genome. ATM subfamily is unique in *A. thaliana*. The LEA_1 subfamily had two members. The LEA_2 subfamily had 46 members. The LEA_3 subfamily, LEA_4 subfamily and LEA_5 subfamily had only one member. In addition, the phylogenetic tree constructed with the 61 PnoLEA proteins sequences was also divided into seven subfamilies (Additional file 1: Figure S1).

### Chromosomal distribution and expansion of the *PnoLEA* genes

**Fig. 4:**
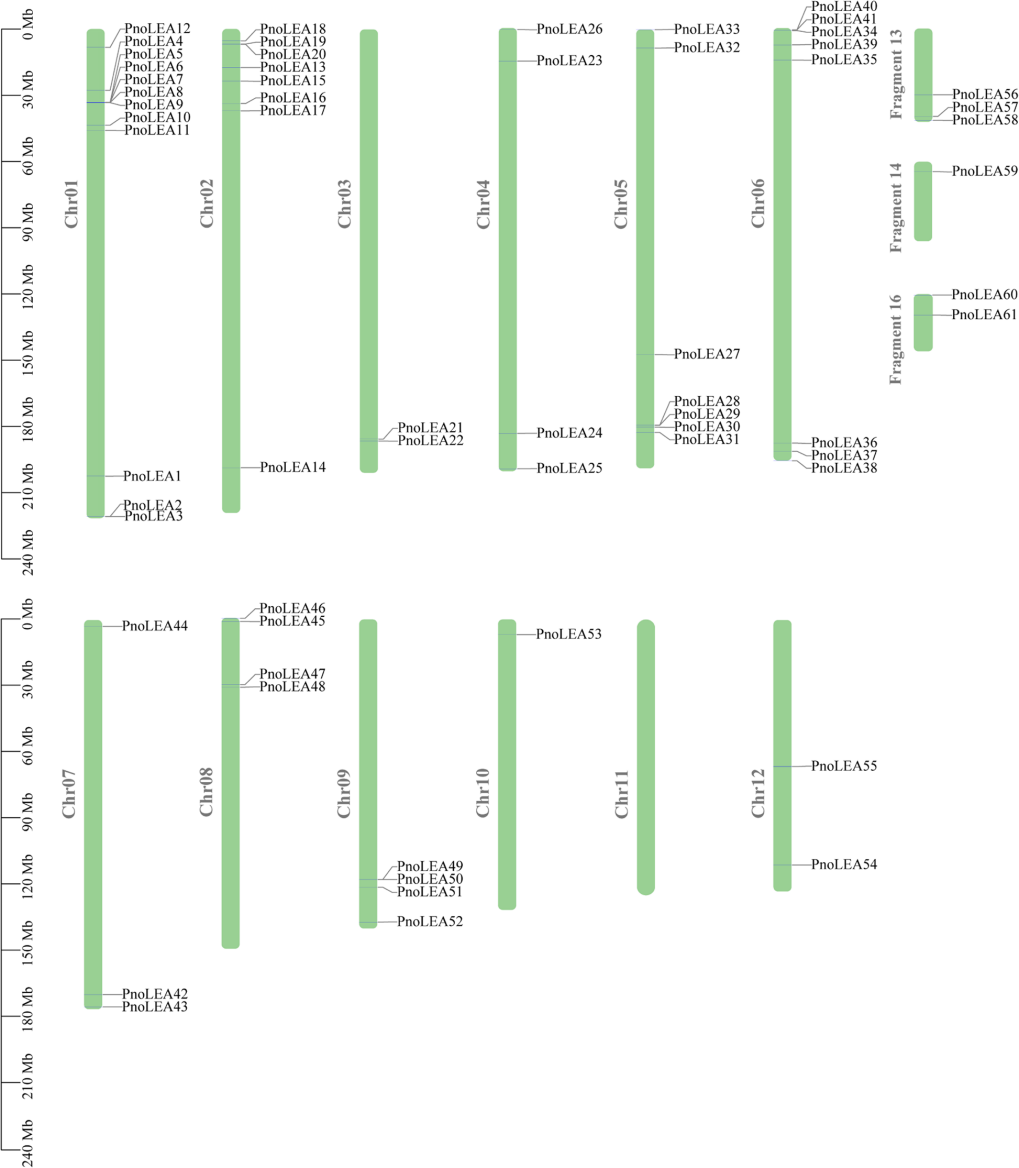
Distribution of the *PnoLEA* genes on chromosomes. The scale of the chromosome is in millions of bases (Mb)

The 61 *PnoLEA* genes were randomly distributed on 11 chromosomes of *P. notoginseng* (Fig. 4). Chromosome 1 has the maximum number of genes, with 12 *PnoLEA* genes. The 8 *PnoLEA* genes were distributed on the chromosome 2 and 6, respectively. The 7 *PnoLEA* genes were distributed on the chromosome 5. There are no genes distributed on chromosome 11 and only one gene was distributed on chromosome 10.

**Fig. 5:**
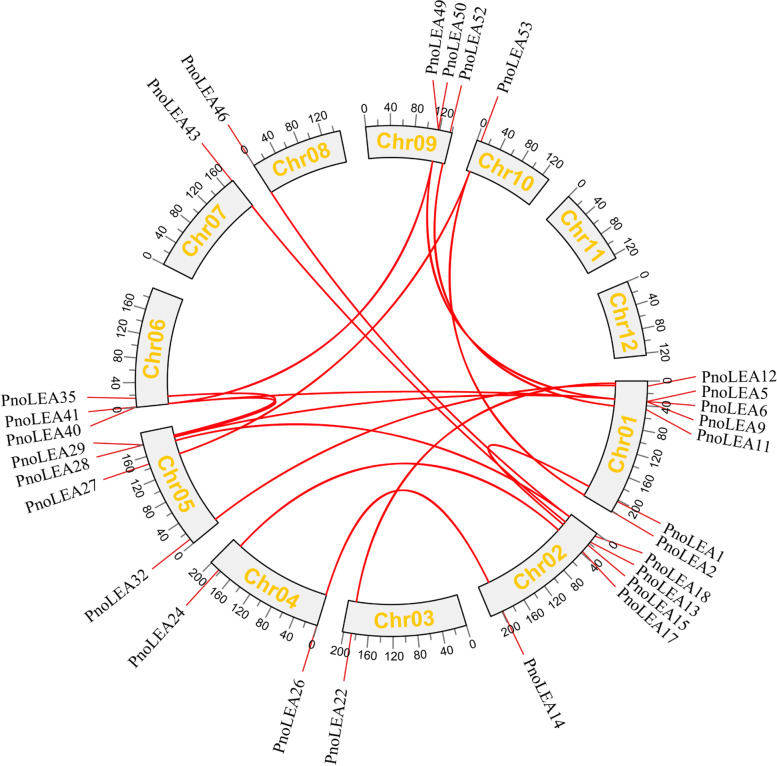
Fragment duplication of *PnoLEA* genes. Fragment duplication of the *PnoLEA* genes are connected by red lines

It is necessary to understand the mechanisms of evolution in the LEA gene family of *P. notoginseng*. We compared the nucleotide sequences of the *PnoLEA* genes in order to confirm their replication patterns. We found 19 pairs of fragment duplication events involving 28 identified homologous genes (Fig. 5). The non-synonymous substitution (Ka) and synonymous substitution (Ks) values of homologous genes pairs were calculated, and Ka/Ks ratios ranged between 0.06 and 0.58. The results showed that these homologous genes might have experienced a purifying selection in the process of evolution (Additional file 2: Table S1). In addition, we compared *PnoLEA* genes with related genes from four species (*A. thaliana*, *Oryza sativa*, *Solanum lycopersicum* and *Zea mays*) (Additional file 3: Figure S2). The results showed that *PnoLEA* genes has more homologues with three dicotyledons (*A. thaliana* and *Solanum lycopersicum*).

### Gene expression analysis of the *PnoLEA* genes in different tissues

**Fig. 6:**
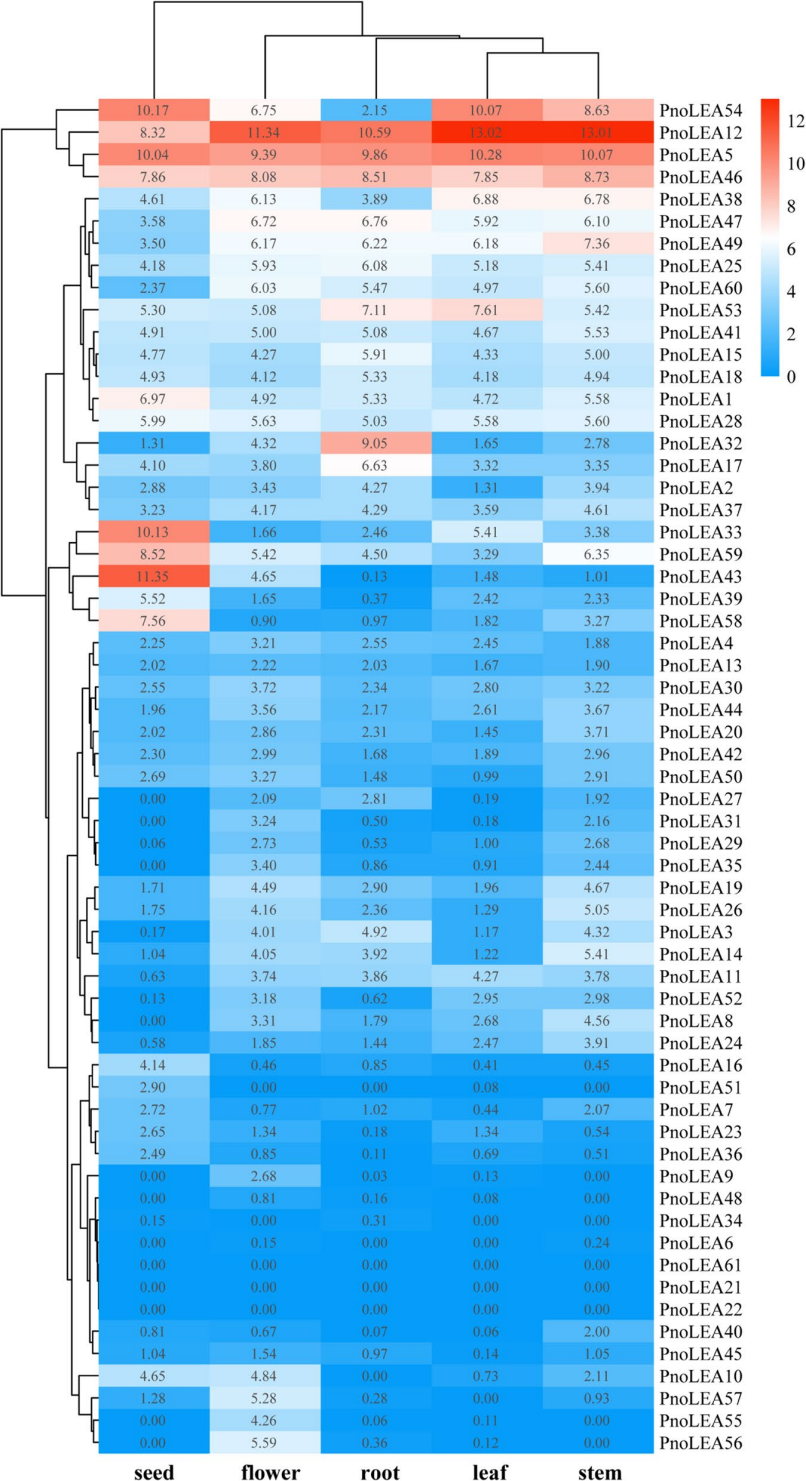
Expression patterns of *PnoLEA* genes in different tissues. The TPM values are transformed using log_2_(TPM + 1)

In order to reveal the tissue specificity of *PnoLEA* genes expression, we selected five tissue types including the roots, stems, leaf, flowers and seeds for detailed transcriptome analysis. The expression of 61 *PnoLEA* genes were divided into two cluster in different tissues and the seeds was divided into one cluster (Fig. 6). The *PnoLEA6*, *PnoLEA21*, *PnoLEA22*, *PnoLEA34*, *PnoLEA48*, and *PnoLEA61* genes were not expressed in five tissues. The expression of *PnoLEA5*, *PnoLEA12*, *PnoLEA46* and *PnoLEA54* genes were up regulated in five tissues. The expression of *PnoLEA33*, *PnoLEA39*, *PnoLEA43*, *PnoLEA58* and *PnoLEA59* genes were up regulated in seeds tissues.

### Changes of germination percentage of *P. notoginseng* seeds during dehydration stress

**Fig. 7:**
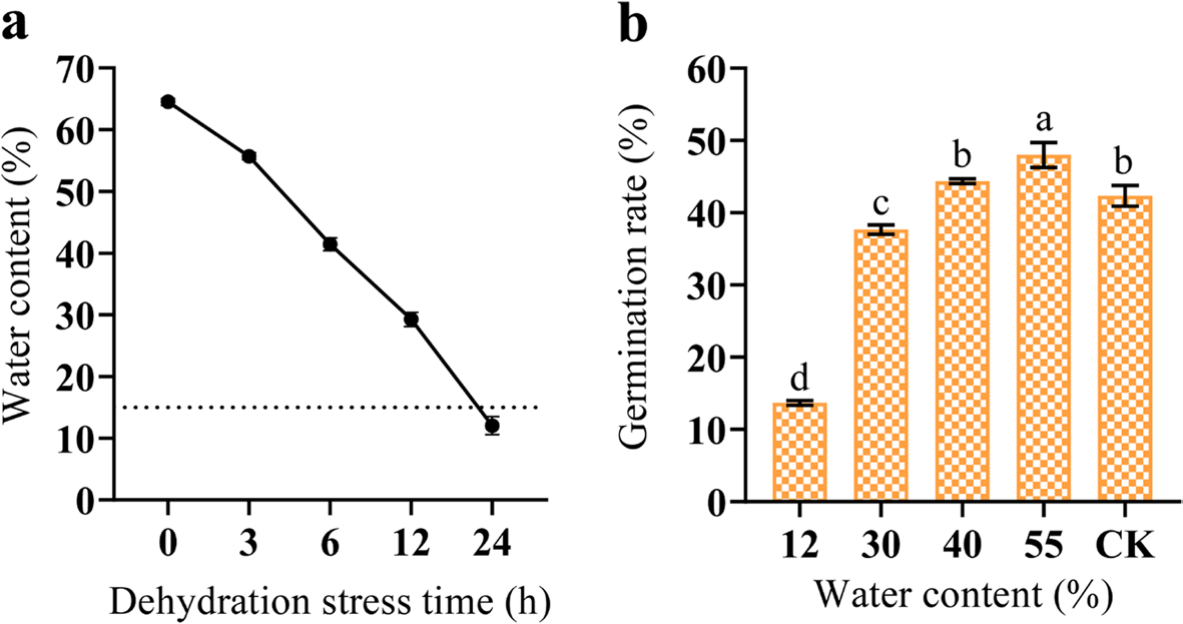
Germination rate of *P. notoginseng* seeds under dehydration stress. **a** Changes of water content. **b** Germination rate of seeds in different water content. Values presented are the means ± SE (*n* = 3). Different letters indicate significant differences among treatments in the same period using Duncan’s test (*P* < 0.05)

The mature seeds of freshly harvested *P. notoginseng* had a high water content of about 64.52% (Fig. 7a). The water content of seeds decreases with increasing dehydration time. After 24 h of dehydration, the water content was below 15% (Fig. 7a). The number of germinations of *P. notoginseng* seeds was slightly increased after a short period (3 h) of dehydration stress (Fig. 7b). The germination rate of *P. notoginseng* seeds was reduced after dehydration stress over 3 h (Fig. 7b). The germination rate of *P. notoginseng* seeds is significantly reduced when the water content of seeds was below 15% (Fig. 7b).

### Expression patterns of *PnoLEA* genes in response to dehydration stress

**Fig. 8:**
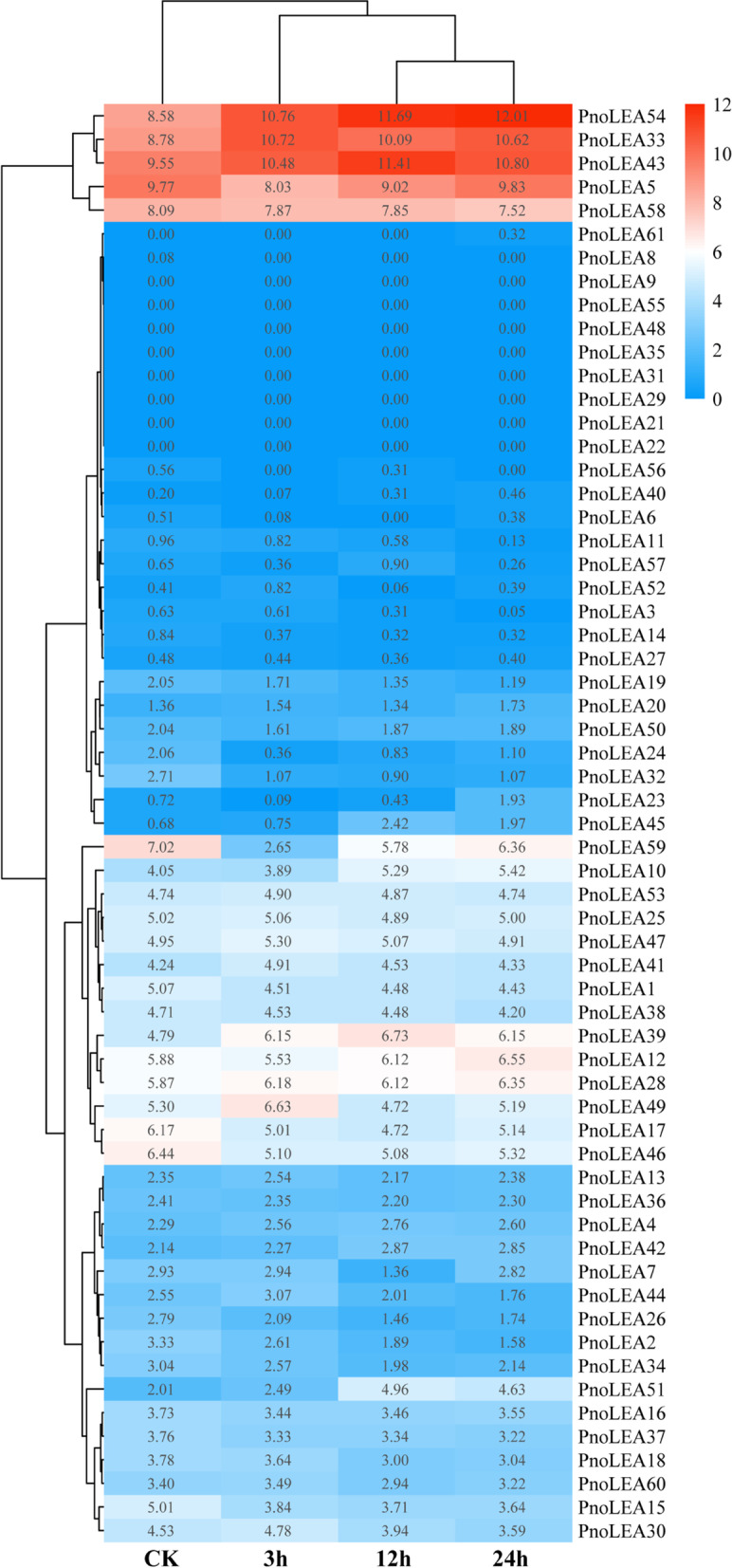
Expression patterns of *PnoLEA* genes in *P. notoginseng* seeds under different dehydration stress. The TPM values are transformed using log_2_(TPM + 1)

The expression of 61 *PnoLEA* genes were divided into three cluster under different levels of dehydration stress and only few genes responded strongly to dehydration stress (Fig. 8). The *PnoLEA54*, *PnoLEA33*, *PnoLEA43*, *PnoLEA5*, and *PnoLEA58* genes were highly expressed under dehydration stress. The second cluster of genes showed lower expression and did not responded to dehydration stress. The expression of the third cluster of genes was slightly or not responsive to dehydration stress.

**Fig. 9:**
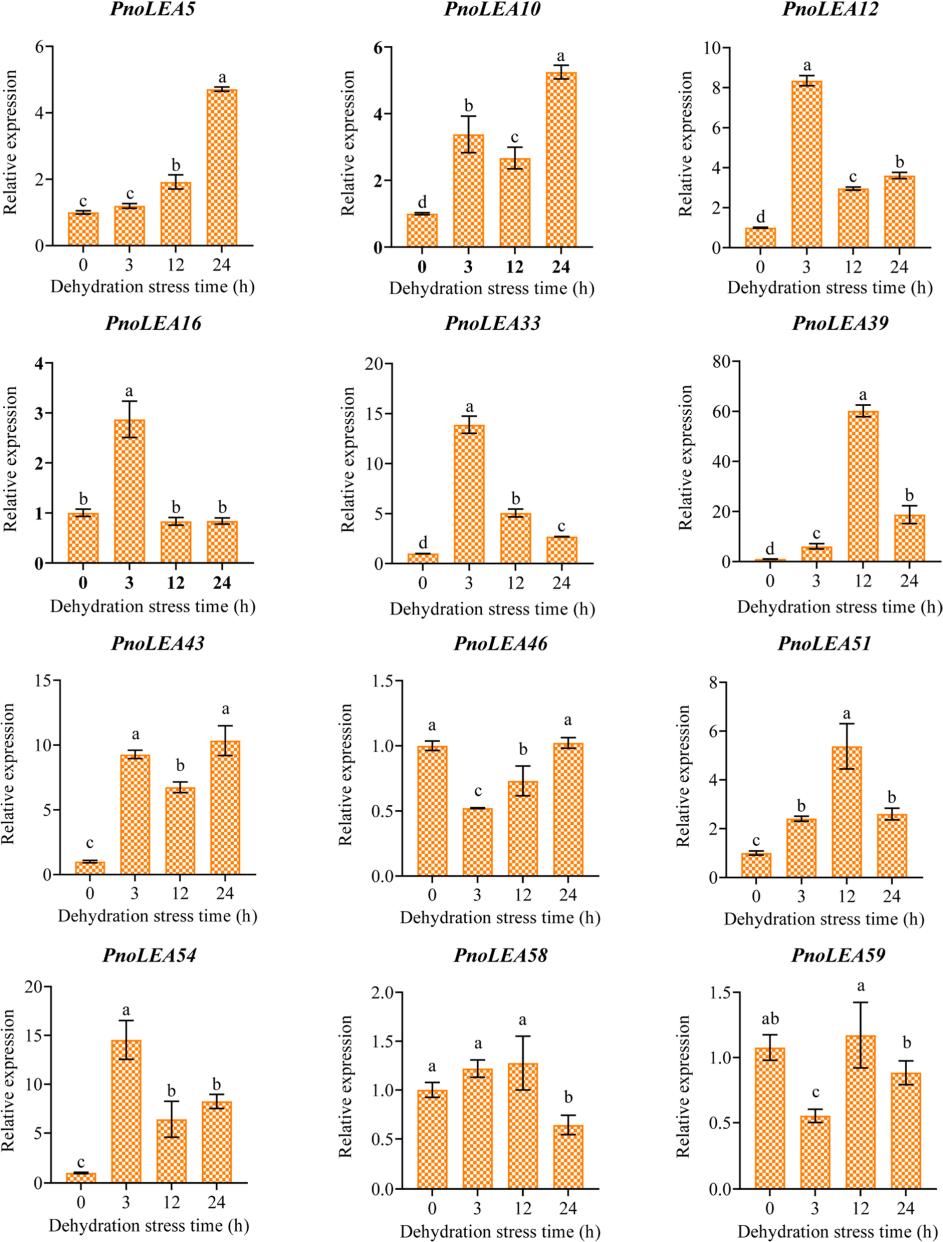
Relative expression of LEA genes in *P. notoginseng* seeds under dehydration stress. Values presented are the means ± SE (*n* = 3). Different letters indicate significant differences among treatments in the same period using Duncan’s test (*P* < 0.05)

To verify the response of *PnoLEA* genes to dehydration stress, we selected genes in each subfamily of LEA gene family that were highly expressed in seeds and validated expression changes using qRT-PCR (Fig. 9). Among them are genes of the LEA_1 subfamily (*PnoLEA10*), the LEA_2 subfamily (*PnoLEA5*, *PnoLEA46*, *PnoLEA58*, *PnoLEA59*), the LEA_4 subfamily (*PnoLEA51*), the LEA_5 subfamily (*PnoLEA33*), the SMP subfamily (*PnoLEA16*, *PnoLEA39*), and the DHN subfamily (*PnoLEA12*, *PnoLEA43*, *PnoLEA54*). Except *PnoLEA48* of the LEA_3 subfamily because its expression was too low. It was observed that the relatively high expression of *PnoLEA12*, *PnoLEA33*, *PnoLEA39*, *PnoLEA43* and *PnoLEA54* under dehydration stress.

## Discussion

### Molecular characteristics of *PnoLEA* genes

The LEA gene family is widely important for plant growth and development [40]. In many species, LEA gene family has been identified, for example, 51 members in *A. thaliana* [5], 27 members in tomato [41], 68 members in *Sorghum bicolor* [42] and 281 members in wheat [43]. In our study, HMM model and BLAST were used to search for *PnoLEA* gene family in the genome database of *P. notoginseng*, and consequently 61 *PnoLEA* genes were identified (Table 1). The numbers of *PnoLEA* genes identified in *P. notoginseng* are consistent with the ones in *S. miltiorrhiza* [11], but is less than the ones in wheat [43] and poplar (*Populus simonii*) [44]. The number of LEA gene family members showed significant differences among species, and this may be related to the ploidy of the species and the expansion of the gene family.

Low intron numbers of genes accelerate its process of transcriptional expression, and it is convenient to decrease the cost for transcription and make cell a fast reaction to abiotic stresses [45]. The 66.7% of *AtLEA* genes in *A. thaliana* contain only one intron [5], and the 62% of the LEA genes in wheat are no introns [43]. Low numbers of introns were also observed in other stress-responsive genes, the most *HSP20* genes (92.6%) are no introns in apple (*Malus domestica*) [46]. Likewise, our results showed that the 51% of *PnoLEA* genes were no intron, 75% of *PnoLEA* genes were 0 ~ 1 intron, and only 6% of *PnoLEA* genes were more than 2 introns, and it could contribute to transcriptional regulation of *PnoLEA* genes in response to stress conditions (Fig. 1). Different groups of LEA proteins show a low similarity [47]. Motif analysis in *A. thaliana* [5], flax (*Linum usitatissimum*) [48] and wheat [49] indicated that the members of LEA gene subfamily contain specifically conserved structures. In our study, the conserved motifs of PnoLEA proteins were different among subfamily groups (Fig. 2), suggesting that PnoLEA proteins probably have specific group functions.

In the PFAM database, the LEA gene family is divided into eight subfamilies [12]. In our study, the LEA_6 subfamily was absent and the 61 *PnoLEA* genes were classified into seven subfamilies (Table 1 and Fig. 3). Consistently, it has been reported that the LEA_6 subfamily is not present in the recalcitrant seeds of tea plants [50]. The LEA_6 subfamily has not been identified in algal and rice in the genomes [51, 52]. Similarly, the LEA_6 subfamily is not identified in the whole genomes of tomato and *Salvia miltiorrhiza* [11, 41], suggesting that the loss events of LEA_6 subfamily may occur during the evolution of these plants. DNA segmental duplication, tandem duplication, and conversion events promote an evolution amplification of the gene family [53]. Among of the *PnoLEA* genes subfamilies, the LEA_2 subfamily has the greater number of members, with 46 (75.4%) members (Table 1 and Fig. 3). This is line with a series of previous study that a large number of LEA_2 subfamily members are found in potato (*Solanum tuberosum*) [54], *Sorghum bicolor* [42], wheat [43], rice [51] and poplar [44]. However, the number of LEA_2 subfamily is low in *A. thaliana* [5] and flax [49]. These results indicate that the LEA gene family has been expanded during the evolutionary process of *P. notoginseng*. Similarly, fragment duplication events are also present in the *PtrLEA* gene family and may contribute to the expansion of the *PtrLEA* genes [44]. Five pairs of homologous genes pairs are identified in cassava (*Manihot esculenta Crantz*), indicating that fragment duplication may be one of the reasons for the expansion of the *MeLEA* gene family in the process of evolution [6]. It has been observed that within 19 pairs of fragment duplication events, among them 17 pairs are LEA_2 subfamily genes (Fig. 5). This result suggested that the expansion of the *PnoLEA* gene family might be caused by the fragment duplication. In addition, the ka/ks ratios of most homologous genes pairs were less than 1. This result suggests that the *PnoLEA* genes may have experienced purifying selection in the process of evolution. Thus, it speculates that the lack of the LEA_6 subfamily and the expansion of the LEA_2 subfamily might be involved in the formation of seed recalcitrance.

### Expression patterns of *PnoLEA* genes and its response to dehydration stress

The LEA genes are expressed widely in plant flower, fruit, seeds, leaf, stems and roots. Seven *MeLEA* genes are low levels in different tissues of cassava, and the expression of eight, seven and five *MeLEA* genes are up regulated in storage roots, stems and leaf, respectively [6]. The expression of the *StASR-2*, *StLEA 1–14*, *StLEA 2–29*, *StLEA 3–3* and *StDHN-3* are up regulated in different tissues of potato, whereas the *StLEA1* and *StSMP* subfamilies are low expressed [54]. The expression of the *PnoLEA* genes in different tissues was analyzed by hierarchical clustering using publicly available RNA-seq data (Fig. 6). The expression of five genes (*PnoLEA5*, *PnoLEA12*, *PnoLEA46* and *PnoLEA54*) were up regulated in all tissues (Fig. 6). Seed tissue was a single cluster in the hierarchical cluster analysis of the different tissues, and five genes (*PnoLEA33*, *PnoLEA39*, *PnoLEA43*, *PnoLEA58* and *PnoLEA59*) specifically expressed in seeds. These results suggest that the expression of *PnoLEA* gene family members is tissue specificity. 93 of 121 *TaLEA* genes are highly expressed in the grain of wheat, and among them there were 35 out of 47 members in the DHN subfamily [55]. There are 50 LEA genes in the flax genome, of which 42 *LuLEA* genes are expressed in all stages in Heiya No.14. However, the number of the expressed *PnoLEA* genes was small in seeds, and only 8 genes were expressed at high levels in the seeds (Fig. 6). We presume that the low transcriptional levels of most *PnoLEA* genes might be associated with the recalcitrance of *P. notoginseng* seeds.

The LEA genes are widely responded to abiotic stresses in plants, such as low temperature, drought and salt stress [40]. Overexpression of *OsEml* gene in rice plants increase osmotic tolerance, when rice plants face with drought stress [1]. The expression of the LEA_1 subfamily, LEA_2 subfamily, LEA_4 subfamily and DHN subfamily genes is up regulated in tomato to respond to drought and salt stress treatments [41]. The genes of the LEA_1 subfamily, LEA_3 subfamily, LEA_4 subfamily and DHN subfamily in *A. thaliana* are also responded to drought stress [56]. Similarly, LEA proteins are abundantly accumulated under dehydration stress in maize seeds [30]. The *CsLEA* genes of tea tree are highly expressed in response to dehydration stress [8]. In our study, the expression of a few genes was up regulated in response to dehydration stress (Fig. 8). The *PnoLEA46*, *PnoLEA58* and *PnoLEA59* genes of LEA_2 subfamily were high expression in seeds, but their expression levels were decreased under dehydration stress (Fig. 8 and Fig. 9). The expression of *PnoLEA43* and *PnoLEA54* genes of the DHN subfamily in seeds is increased under dehydration stress. Previous study have shown that the alpha helix structure of DNH protein is amphiphilic and binds to the plasma membrane to prevent cell dehydration [57]. These results suggest that the DNH subfamily plays an important role in response to dehydration stress in *P. notoginseng* seeds and the expansion of the LEA 2 subfamily may lead to its non-functionalization or functional divergence. An increase in the germination rate of *P. notoginseng* seeds was observed after a short period of dehydration stress, while it was decreased with increasing time of dehydration stress (Fig. 7). The result is consistent with the previous studies that appropriate dehydration stress could promote the germination of recalcitrant seeds [58, 59]. The germination rate of recalcitrant *Quercus wutaishanica* seeds was increased with light dehydration stress and decreased with increasing time of dehydration stress [59]. The expression of the *PnoLEA33* (SMP) was significantly increased under appropriate dehydration stress treatment (Fig. 8 and Fig. 9). We believe that the increase in the expression the *PnoLEA33* gene might promote the accumulation of seed maturation proteins, thus increasing germination rate of *P. notoginseng* seeds. We presume that the protective effect of the LEA proteins might be limited, and cells have been irreversibly damaged at the critical water content (15%) of *P. notoginseng* seeds. These evidences confirm that LEA proteins are essential in seed germination and the response of recalcitrant seeds to dehydration stress, thus providing new insights into recalcitrant seeds in agricultural production and storage.

## Conclusions

In summary, we identified 61 *PnoLEA* genes from *P. notoginseng* genome. They were divided into seven subfamilies based on phylogenetic relationships, gene structures and protein conserved domains. Most members of the *PnoLEA* genes family show a low number of introns, and this could be related to a rapid response to dehydration stresses. We believe that the lack of the LEA_6 subfamily, the expansion of the LEA_2 subfamily and low transcriptional levels of most *PnoLEA* genes might be involved in the formation of seed recalcitrance of *P. notoginseng*. LEA proteins are critical in the response to dehydration stress in recalcitrant seeds, and the protective role of LEA proteins is closely related to the degree of recalcitrance of the seeds. These findings improve our understanding of the role of LEA proteins in the response of recalcitrant seeds to dehydration stress.

## Methods

### Genome-wide identification of the *PnoLEA* genes

The genomic data of *P. notoginseng* was obtained in the Herbal Medicine Omics Database (http://herbalplant.ynau.edu.cn) [60]. The Hidden Markov Model (HMM) profiles of LEA (PF00257, PF00477, PF02987, PF03168, PF03242, PF03760, PF04927 and PF10714) were downloaded from the InterPro database (https://www.ebi.ac.uk/interpro). The local genome database of *P. notoginseng* proteins were scanned using the HMMER [61]. LEA proteins of *A. thaliana* were downloaded from the database TAIR (https://www.arabidopsis.org), and the sequence of the AtLEA proteins as reference sequences was blasted search in the protein sequence of *P. notoginseng*. The original candidate LEA proteins of *P. notoginseng* were obtained by combining BLAST and HMMER results. The SMART tool was used to check for conserved domains in the LEA protein sequences of the original candidate (http://smart.embl-heidelberg.de), and the protein sequences lacking the LEA domains were removed. The Expasy website (https://web.expasy.org) was used to calculate the isoelectric point (pI) and molecular weight of LEA proteins. The BUSCA annotation system was used to predict the subcellular localization of LEA proteins (https://busca.biocomp.unibo.it).

### Phylogenetic and gene structure of *PnoLEA* genes

The LEA proteins of *P. notoginseng* and *A. thaliana* were performed by multiple alignment of full-length amino acid sequences using the MATTF program [62]. The phylogenetic tree was constructed by the MEGA11 program using the neighbor joining method with 1000 bootstrap replicates [63]. The exons and introns of sequences were examined, and visualized with the R. The MEME Suite was used to predict the conserved motifs of the PnoLEA protein [64].

### Chromosomal distribution and gene duplication of the *PnoLEA* genes

We used TBtools software to map the distribution of the *PnoLEA* genes on the chromosome [65]. MCScanX program was used to calculate duplicate events for the *PnoLEA* genes [66]. The KaKs_Calculator software was used to calculate the ratios of non-synonymous substitution and synonymous substitution (Ka/Ks) for duplication genes pairs in *P. notoginseng* [67]. In addition, the genomic sequences of four species (*A. thaliana*, *Oryza sativa*, *Solanum lycopersicum* and *Zea mays*) were acquired in the Ensembl Plants database (https://plants.ensembl.org/index.html). The collinearity of *LEA* homologous genes between *P. notoginseng* and the four species analyzed using MCScanX, and visualized with the Dual Synteny Plotter of TBtools software [65].

### Plant material and dehydration stress

The *P. notoginseng* of three-year-old was planted to be used as experimental material in experimental fields in Wenshan Miao Xiang, Yunnan Province, China. The mature seeds of *P. notoginseng* were picked in November. Seeds were removed the red peel, disinfected with 5% CuSO_4_ for 30 min, washed with double distilled water (ddH_2_O) twice, dried indoors shade. The seeds of *P. notoginseng* were dehydrated with silica gelilica gel for 3 h, 6 h, 12 h and 24 h respectively. The ratio was 1:10 between seed weight and silica gel weight, and the silica gel was changed every 6 h. The seeds were placed in well-ventilated fully meshed baskets in a wet sand-laminated indoors at a temperature of 15 ± 5 °C, and kept the water content of the seeds was at about 60%. After 50 days, the number of seed germination was observed.

### Expression analysis of *PnoLEA* genes

The transcriptome data of different tissues of *P. notoginseng* were obtained from public databases. The RNA-Seq data of roots, stems, leaf and flowers of *P. notoginseng* were downloaded from the NCBI public database (Accession Number: SRR7427743, SRR7427744, SRR7427758, SRR7427747, SRR7427748, SRR7427750, SRR7427752, SRR7427755, SRR7427756 SRR7427752, SRR7427755, SRR7427756, SRR7427751, SRR7427754, SRR7427757), and the BioProject related links are https://www.ncbi.nlm.nih.gov/bioproject/PRJNA477910 [68]. The RNA-Seq data of *P. notoginseng* seeds were obtained from the NGDC database (GSA: CRA008378, https://ngdc.cncb.ac.cn).

For 3 h, 12 h, 24 h and CK treatment, the sample used for RNA extraction. Total RNA was extracted using Plant Plus Kit (Tiangen, Beijing, China) according to the manufacturer’s protocol, with three replications. RNA samples were tested for degradation and impurities by using 1% agarose electrophoresis. RNA quality was assessed on an Agilent 2100 Bioanalyzer (Agilent Technologies, Palo Alto, CA, USA) and checked using RNase free agarose gelelectrophoresis. Sequencing libraries were generated using NEBNext® UltraTM RNA Library Prep Kit for Illumina® (NEB, USA) following manufacturer’s recommendations and index codes were added to attribute sequences to each sample. The Illumina HiSeq platform was used to perform cDNA library sequencing and acquire a large amount of high-quality data. The raw data have been submitted to the NGDC database with the GSA number CRA010115.

The raw reads were cleaned using Trimmomatic (version 0.39) to remove low quality reads and reads containing adapter [69]. The expression levels of the genes were calculated using the salmon [70] and transformed using log_2_(TPM + 1) for the mean of three biological replicates. Using the R program pheatmap, the hierarchical clustering of expression levels was visualized.

### Total RNA extraction and qRT-PCR analysis

In order to determine the expression of the *PnoLEA* genes under different levels of dehydration stress and to explain the relationship between LEA protein and dehydration sensitivity, qRT-PCR analysis was performed on the *PnoLEA* genes. The total RNA of *P. notogensing* seeds was extracted using a TAKARA MIniBEST Plant RNA Extraction kit. Using the Prime Script RT kit (Takara Bio, Kyoto, Japan), RNA was reverse transcribed into cDNA. The Premier 3.0 software was used to design the primer sequences of qRT-PCR [71] and the primer sequences were synthesized by Shanghai Generay Biotech Co., Ltd. (Shanghai, China). The primer sequences were showed Table S2. The reference gene used for *P. notoginseng* seeds was *GLYCERALDEHYDE-3-PHOSPHATE DEHYDROGENASE(GAPDH)*. The Quant studio12K Flex System (Thermo Fisher Scientific) was used for qRT-PCR with three technical replicates. The relative expression levels of LEA genes were calculated using the 2^−ΔΔCt^ method [72].

## Supplementary Information

**Additional file 1:Figure S1.** The neighbor-joining (NJ) phylogenetic tree of PnoLEA proteins. *PnoLEA* genes families are grouped by different colors. The tree was constructed with amino acid sequences of identified *PnoLEA* genes and bootstrap value of 1000 replicates.

**Additional file 2:Table S1.** The list of 19 pairs repetitive events in LEA genes of *P. notoginseng* and its Ka/Ks ratio.

**Additional file 3:Figure S2.** Collinearity map of the *PnoLEA* genes in *P. notoginseng* to other four species. The blue lines denote collinearity between the *PnoLEA* genes and other species, while the gray lines represent collinearity between the *P. notoginseng* genome and other species.

**Additional file 4:Table S2.** Primers designed for Quantitative Real-time PCR (qRT-PCR) in *P. notoginseng*.

## Data Availability

The raw RNA-Seq data in different tissues (root, stem, leaf and flower) of *P. notoginseng* are available in the NCBI database under the Bioproject accession number PRJNA477910 (https://www.ncbi.nlm.nih.gov/bioproject/PRJNA477910). All data generated or analyzed during this study are included in this published article and its supplementary information files. The raw sequencing data of *P. notoginseng* seeds for this study have been deposited in the Genome Sequence Archive in BIG Data Center (https://bigd.big.ac.cn/), Beijing Institute of Genomics (BIG), Chinese Academy of Sciences, under the accession number: CRA008378, CRA010115. Other data generated or analyzed during this study are included in this published article and its supplementary information files. Hoo & Tseng firstly undertook the formal identification of the plant material *Panax notoginseng* (Burkill) (Journal of Systematics and Evolution 11: 435, 1973) in Flora of China.

## Abbreviations

Ka: Non-synonymous substitution rate
Ks: Synonymous substitution rate
LEA: Late embryogenesis abundant
qRT-PCR: Quantitative real-time PCR
SMP: Seed maturation protein
TPM: Transcripts per kilobase of exon model per million mapped reads
