# Supplementary material for: Genome-wide identification and characterization of members of the LEA gene family in Panax notoginseng and their transcriptional responses to dehydration of recalcitrant seeds
Source: BMC Genomics. 2023 Mar 17;24:126. doi: 10.1186/s12864-023-09229-0 (PMC10024439; doi:10.1186/s12864-023-09229-0)
Supplement: Supplementary file 1 — Additional file 1:Figure S1. The neighbor-joining (NJ) phylogenetic tree of PnoLEA proteins. PnoLEA genes families are grouped by different colors. The tree was constructed with amino acid sequences of identified PnoLEA genes and bootstrap value of 1000 replicates. [file 12864_2023_9229_MOESM1_ESM.docx]

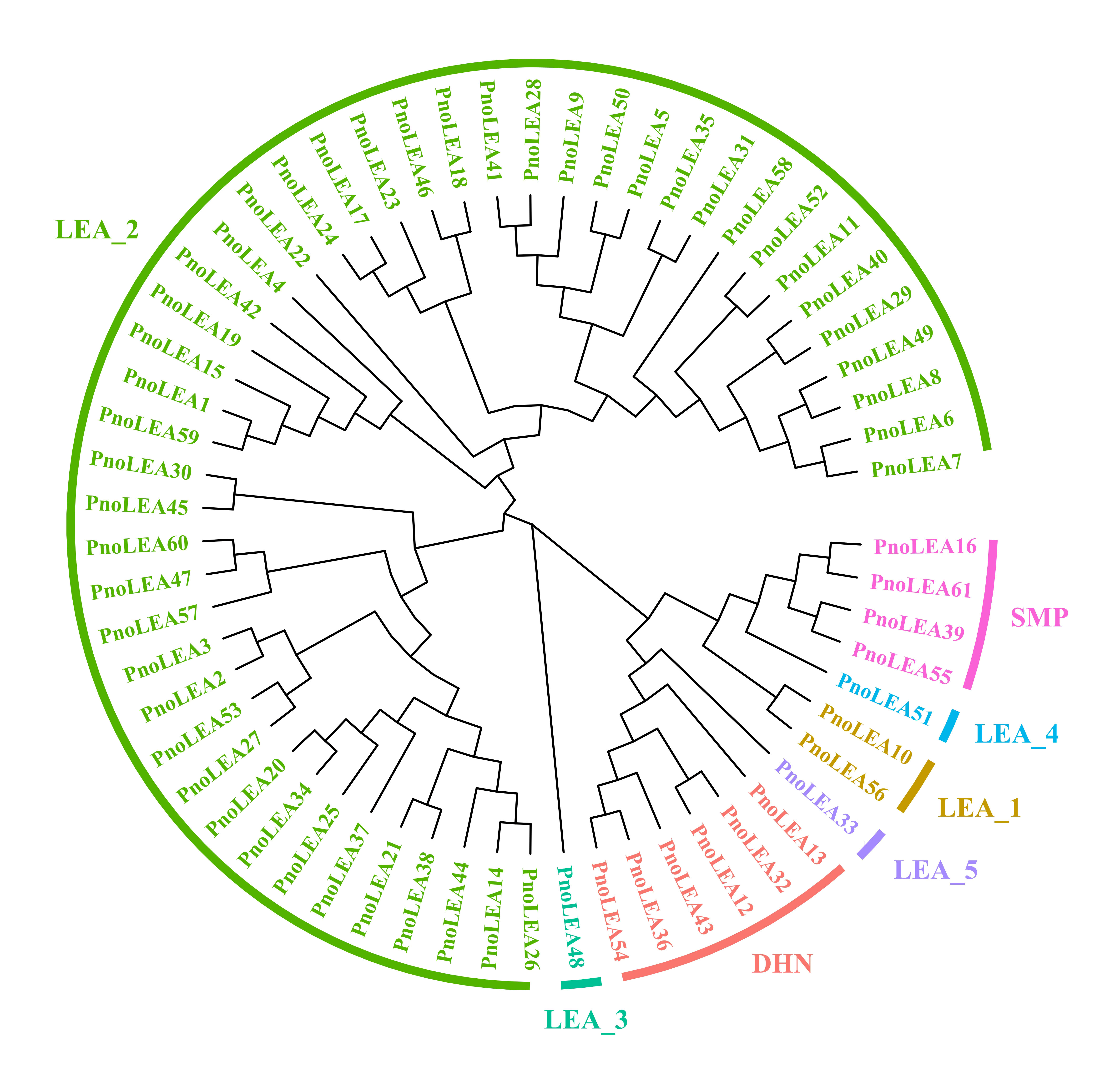


**Additional file 1: Figure S1.** The neighbor-joining (NJ) phylogenetic tree of PnoLEA proteins. *PnoLEA* genes families are grouped by different colors. The tree was constructed with amino acid sequences of identified *PnoLEA* genes and bootstrap value of 1000 replicates.
