# Supplementary material for: Genome-wide identification and characterization of members of the LEA gene family in Panax notoginseng and their transcriptional responses to dehydration of recalcitrant seeds
Source: BMC Genomics. 2023 Mar 17;24:126. doi: 10.1186/s12864-023-09229-0 (PMC10024439; doi:10.1186/s12864-023-09229-0)
Supplement: Supplementary file 3 — Additional file 3:Figure S2. Collinearity map of the PnoLEA genes in P. notoginseng to other four species. The blue lines denote collinearity between the PnoLEA genes and other species, while the gray lines represent collinearity between the P. notoginseng genome and other species. [file 12864_2023_9229_MOESM3_ESM.docx]

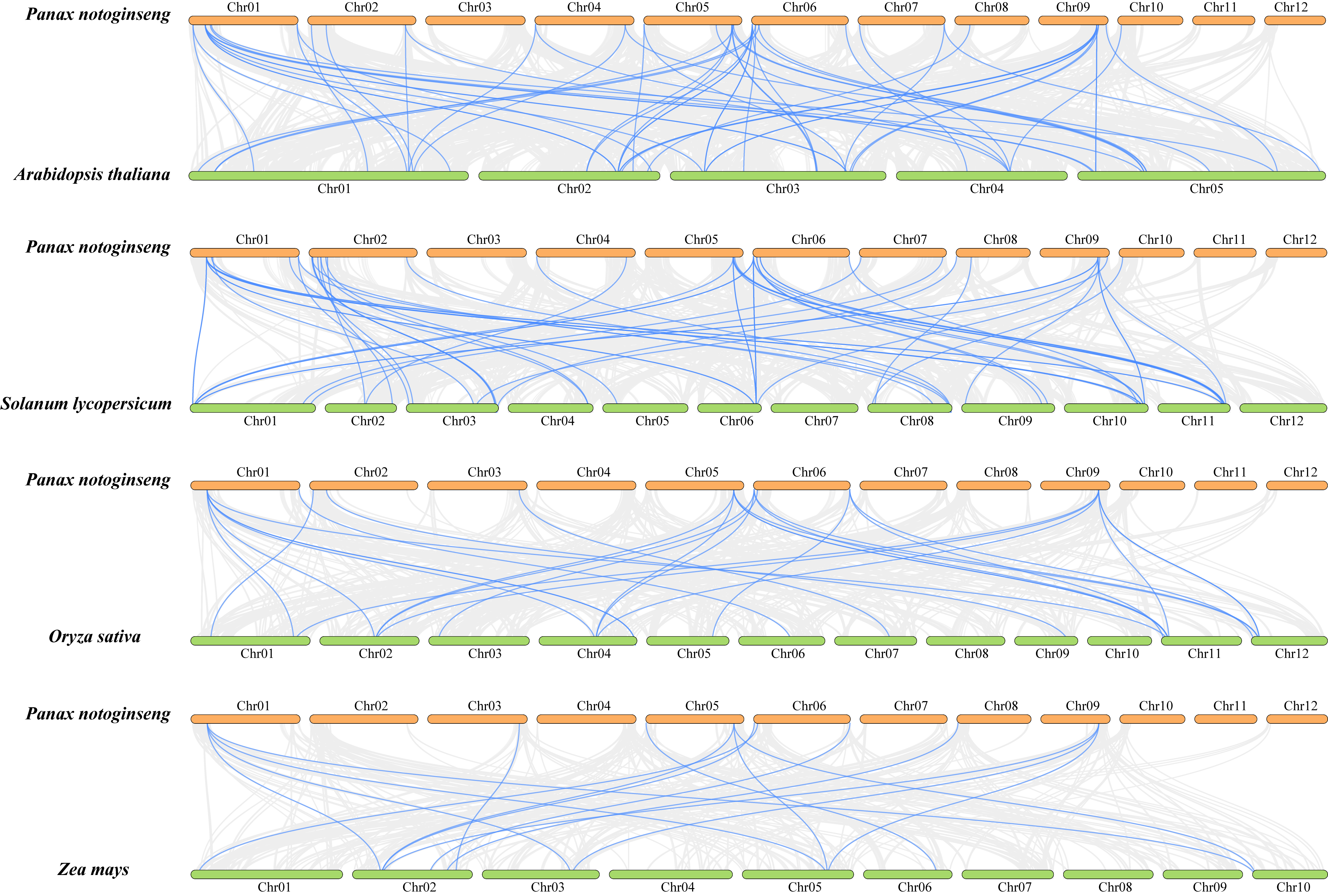


**Additional file 3: Figure S2.** Collinearity map of the *PnoLEA* genes in *P. notoginseng* to other four species. The blue lines denote collinearity between the *PnoLEA* genes and other species, while the gray lines represent collinearity between the *P. notoginseng* genome and other species.
